# Maternal, Infant Characteristics, Breastfeeding Techniques, and Initiation: Structural Equation Modeling Approaches

**DOI:** 10.1371/journal.pone.0142861

**Published:** 2015-11-13

**Authors:** Ying Lau, Tha Pyai Htun, Peng Im Lim, Sarah Ho-Lim, Piyanee Klainin-Yobas

**Affiliations:** 1 Department of Alice Lee Centre for Nursing Studies, Yong Loo Lin School of Medicine, National University of Singapore, Singapore; 2 Nursing Department, National University Hospital, Singapore; 3 Department of Obstetrics and Gynaecology, National University Hospital, Singapore; Liverpool School of Tropical Medicine, UNITED KINGDOM

## Abstract

**Objectives:**

The aim of this study was to examine the relationships among maternal and infant characteristics, breastfeeding techniques, and exclusive breastfeeding initiation in different modes of birth using structural equation modeling approaches.

**Methods:**

We examined a hypothetical model based on integrating concepts of a breastfeeding decision-making model, a breastfeeding initiation model, and a social cognitive theory among 952 mother-infant dyads. The LATCH breastfeeding assessment tool was used to evaluate breastfeeding techniques and two infant feeding categories were used (exclusive and non-exclusive breastfeeding).

**Results:**

Structural equation models (SEM) showed that multiparity was significantly positively associated with breastfeeding techniques and the jaundice of an infant was significantly negatively related to exclusive breastfeeding initiation. A multigroup analysis in the SEM showed no difference between the caesarean section and vaginal delivery groups estimates of breastfeeding techniques on exclusive breastfeeding initiation. Breastfeeding techniques were significantly positively associated with exclusive breastfeeding initiation in the entire sample and in the vaginal deliveries group. However, breastfeeding techniques were not significantly associated with exclusive breastfeeding initiation in the cesarean section group. Maternal age, maternal race, gestations, birth weight of infant, and postnatal complications had no significant impacts on breastfeeding techniques or exclusive breastfeeding initiation in our study. Overall, the models fitted the data satisfactorily (GFI = 0.979–0.987; AGFI = 0.951–0.962; IFI = 0.958–0.962; CFI = 0.955–0.960, and RMSEA = 0.029–0.034).

**Conclusions:**

Multiparity and jaundice of an infant were found to affect breastfeeding technique and exclusive breastfeeding initiation respectively. Breastfeeding technique was related to exclusive breastfeeding initiation according to the mode of birth. This relationship implies the importance of early effective interventions among first-time mothers with jaundice infants in improving breastfeeding techniques and promoting exclusive breastfeeding initiation.

## Introduction

Breastfeeding is nationally promoted as the ideal method of infant nutrition due to its numerous benefits to mothers, children, and communities [1,2]. According to the United Nations Fund for Children, optimal infant breastfeeding should be initiated within the first hour of birth, then exclusive breastfeeding continues for 6 months, and appropriate complementary feeding will commence after the 6^th^ month together with breastfeeding for at least 2 years [3]. In fact, long-term breastfeeding depends on exclusive breastfeeding initiation in early postpartum [4]. It is widely recognized that breastfeeding is a learned skill because breastfeeding is not a single suckling action but a series of behaviors which depends on the integrated coordination between mothers and infants [5]. However, low rates of breastfeeding initiation and early cessation of breastfeeding are prevalent in many industrialized countries, including Singapore [6]. Different modes of birth, in particular cesarean section, are widely believed to affect early breastfeeding adversely [7]. Cesarean section is a commonly performed surgical procedure which the World Health Organization (WHO) proposed should not exceed 15% of all births [8]. It accounts for 15% of all deliveries globally [9,10] and it is even more widespread in Asia, where the cesarean section rate is 27.3% [11]. However, its incidence has increased rapidly worldwide over the last two decades [12] and it is no exception in Singapore, where it saw a significant increase in cesarean section rate from 19.9% in 2000 to 29.6% in 2010 [13].

Although breastfeeding is a natural phenomenon, successful breastfeeding can be a complex task for the mother-infant dyad. Several factors can be used to measure breastfeeding effectiveness, including the mother's correct positioning of her infant at the breast, her comfort level, type of nipple, infant feeding techniques, such as rooting, latching, active sucking, and audible swallowing [14–17], all of which was found in these studies to be objective predictors of successful breastfeeding. However, cesarean section can negatively influence breastfeeding initiation and techniques due to mothers’ mobility limitations, positioning difficulties, post-surgical pain and discomfort, and separation of mother and infant in the first days after birth [18,19]. In addition, the analgesia administered to mothers for pain relief after cesarean section can impact infants’ ability to latch on their mothers’ breast [14]. Consequently, a study has also found that mothers who had just delivered their infants through cesarean section found breastfeeding to be more stressful than mothers who had vaginal deliveries [20].

Given that women’s decisions on initiating exclusive breastfeeding differ according to the modes of birth, it is important to investigate maternal and infant characteristics associated with breastfeeding techniques separately among the entire sample, and in the cesarean section and vaginal delivery groups. This relationship provides knowledge that will help health-care providers improve breastfeeding outcomes in the different modes of birth. Maternal and infant factors associated with breastfeeding techniques include age [21], race [22], parity [21], gestation [23], birth weight [21], and jaundice [24]. Younger mothers (< 20 years old) demonstrated poor positioning of their infants compared with other age groups [21]. Race, due to multiferous cultural norms, is one of the factors influencing breastfeeding [22]. Having more children has been significantly associated with better breastfeeding skills [21]. Preterm infants are at increased risk of feeding difficulties [23] and low birth weight infants have been found to be significantly associated with poor attachment and suckling [21]. A study also found that jaundiced infants experiencing bouts of lethargy may experience difficulties latching on and suckling [24]. In addition, women who experienced postnatal complications may increase the risk of difficulty with breastfeeding techniques or initiation [25,26]. Given this evidence, we were curious to know if there were any differences between the cesarean section and vaginal delivery groups in regards to maternal and infant characteristics in breastfeeding techniques and exclusive breastfeeding initiation.

### Conceptual Framework and Hypothetical Model

A hypothesized model was established by integrating the concepts of a breastfeeding decision-making model [27], a breastfeeding initiation model [28], and a social cognitive theory [29]. This model posits that determinism exists among factors that function in a dynamic fashion to influence health behavior. Maternal and infant characteristics may be underlying factors affecting breastfeeding techniques [27]. Breastfeeding techniques that were assessed by the LATCH breastfeeding assessment tool [30] included five areas (latching, swallowing, types of nipple, comfort level, and positioning [30]) that affect exclusive breastfeeding initiation [31–33].

**Fig 1:**
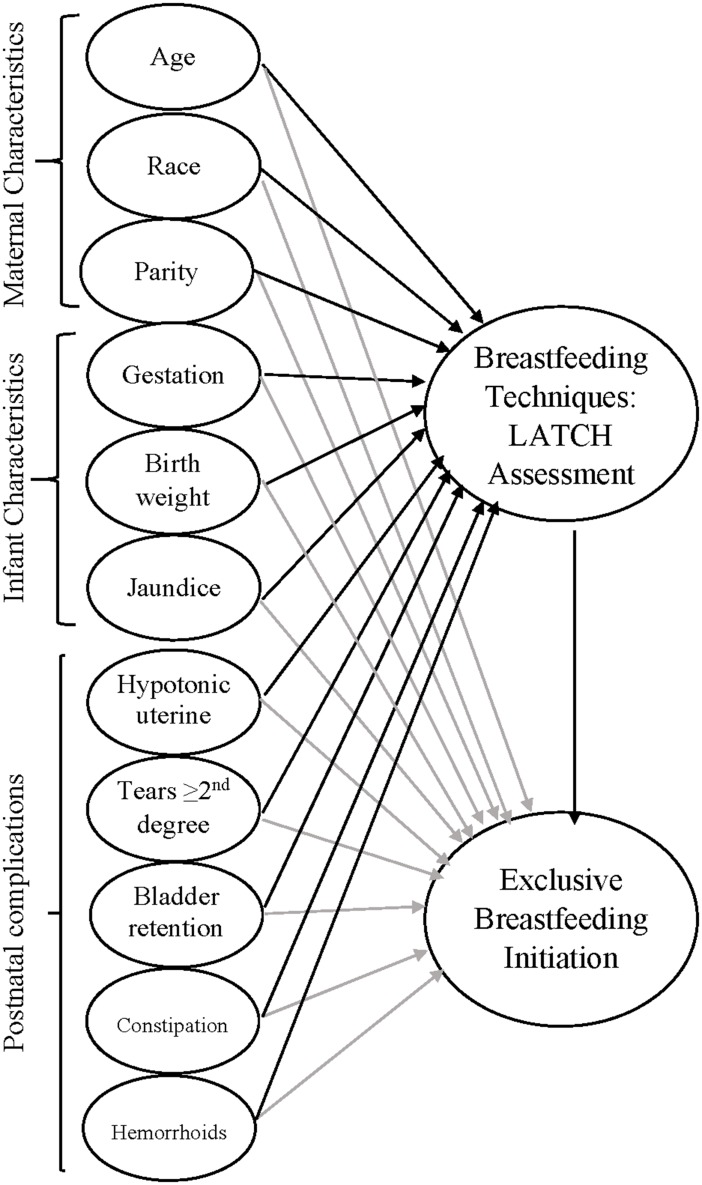
A hypothetical model.

There is both empirical and theoretical support for the relationships among maternal and infant characteristics, postnatal complications, breastfeeding techniques, and exclusive breastfeeding initiation. Therefore, we proposed a hypothetical model (Fig 1).

To our knowledge, only a few studies have examined maternal and infant characteristics, breastfeeding techniques, and exclusive breastfeeding initiation in different modes of birth using structural equation modeling (SEM) approaches. The SEM allows us to study real-life phenomenon using a statistical procedure to link the philosophy of science to theoretical and empirical research [34]. Thus, the current study was designed to address this possibility by providing theoretical, empirical, and practical insights into the conditions under which maternal and infant characteristics are linked to breastfeeding techniques and exclusive breastfeeding initiation in different modes of delivery. The findings of this study can extend our understanding of breastfeeding and delivery methods in terms of their relationships. Given the need for more information on these relationships, the following two research questions were asked:

1. What are the associations between each of the following: maternal characteristics (maternal age, race, parity), infant characteristics (gestation, birth weight, jaundice), and postnatal complications (hypotonic uterine, tears ≥ 2^nd^ degree, bladder retention, constipation, hemorrhoids) on breastfeeding techniques?
2. What are the associations between each of the following: maternal characteristics (maternal age, race, parity), infant characteristics (gestation, birth weight, jaundice) and postnatal complications (hypotonic uterine, tears ≥ 2^nd^ degree, bladder retention, constipation, hemorrhoids) on exclusive breastfeeding initiation?
3. Do breastfeeding techniques predict exclusive breastfeeding initiation?

## Methods

### Design

This is an exploratory cross-sectional quantitative design.

### Setting and Sampling

Singapore is a multiethnic country in Southeast Asia with a population of more than 5.39 million people covering an area of 716.1 km^2^ [35]. The participants of this study were recruited from a tertiary hospital with a delivery rate of 2935 deliveries/year. This regional public hospital provides comprehensive obstetric services to women and children of different demographic and socio-economic groups. Convenience sampling was used due to resource constraint. The sample size was determined by power analysis for the SEM [36]. Given the number of latent variables = 5 and number of observed variables = 8 in this study, the minimum sample size required was 508 to achieve a power of 0.80, an effect size of 0.3, and a probability level of 0.05 [37]. The inclusion criteria for the participants were postnatal women who delivered babies in two postnatal wards. Exclusion criteria included (1) maternal severe psychiatric illnesses and physical disabilities, (2) women with human immunodeficiency virus infection, (3) women with major breast surgery preventing establishment of effective breastfeeding, and (4) infants transferred to neonatal intensive care units with congenital abnormalities.

### Data Collection

The study was reviewed and approved by the hospital ethics committee (Reference No: 2013/00513). Women who delivered babies during hospitalization in two postnatal wards within the data collection period of September 2013 to August 2014 were screened. Each eligible postnatal woman was invited to participate in the study. A full explanation of the study was given using a patient’s information sheet. Participation in the study was voluntary and the respondents’ anonymity was assured. The completed questionnaires were collected with no identifiers of the participants.

### Instruments

Maternal characteristics (age, race, and parity), infant characteristics (gestations, birth weight, and jaundice), exclusive breastfeeding initiation, and postnatal complications (hypotonic uterine, ≥ 2^nd^ tears, bladder retention, constipation, and hemorrhoids) were collected from the participants’ medical records. The postnatal nurse staff assessed the five parameters of LATCH (latching, swallowing, type of nipple, comfort level, and positioning) for participants within 48 to 72 hours after delivery. Exclusive breastfeeding (either feeding at the breast or feeding with expressed breast milk) [38] initiation was assessed whether breastfeeding was initiated within 1 hour to 72 hours after delivery during hospitalization (i.e. ‘yes’ versus ‘no’). Incident neonatal jaundice was based on visual assessment of the infant and was reported either as “yes” or “no” at routine examination before discharge from the hospital.

The LATCH breastfeeding assessment tool [30] was used to evaluate breastfeeding techniques based on observations and descriptions of effective breastfeeding. The letters of the acronym, LATCH, designate separate areas of assessment: “L” for how well the infant latches onto the breast; “A” for the amount of audible swallowing; “T” to describe the type of the mother’s nipple; “C” for the mother’s level of comfort; and “H” for the amount of support the mother has given to hold her infant to the breast. The tool assigned a numerical score—0, 1, or 2—for these five areas, with “0” scored for poor latching (L), ineffective swallowing (A), inverted nipple (T), engorged breast or cracked nipple (C), or poor positioning (P), and “2” scored for good latching (L), audible swallowing (A), everted nipple (T), soft and tender breast (C), and good positioning (H). The total score ranges from 0 to 10, with a higher score representing better breastfeeding techniques. The score also reflects the degree of assistance the hospital staff would provide for mother-infant pairs during breastfeeding by assigning priorities in the provision of breastfeeding assistance. It is a systematic documentation and standardized communication tool used among health-care professionals [30] and such a tool can assist health-care professionals to assess breastfeeding knowledge and skills [39]. Psychometric properties tests for the LATCH assessment tool have demonstrated satisfactory inter-rater reliability [39–41], construct validity [40], concurrent validity [41], and predictive validity [42,43].

### Data analysis

IBM SPSS Statistics 22.0 (IBM Corporation, Armonk, NY, USA) was used for data analysis and missing data were treated with mean substitution. Descriptive statistics were used to analyze the maternal characteristics, infant characteristics, breastfeeding techniques, exclusive breastfeeding initiation, and postnatal complications. Subgroups of mother-infant dyads and the mean differences of the LATCH scores were compared by the chi-square (χ^2^) and independent *t* test. The SEM was performed with the Analysis of Moment Structures (AMOS) software (version 22.0) to test structural relationships among predictors and dependent variables [44]. Its ability lies in the assessment of latent variables at observation level and testing hypothesized relationships between latent variables at theoretical level [34], hence the SEM was the preferred analytic strategy for analyzing the relationships among the constructs (maternal characteristics, infant characteristics, postnatal complications, breastfeeding techniques, and exclusive breastfeeding initiation) simultaneously in our hypothetical model [45].

To test the proposed hypothetical models based on our theoretical assumption [27,28], a two-step approach was used. Firstly, confirmatory analysis was used to assess how well the observed measures (five subscales of LATCH assessment tool) reflected the latent constructs (breastfeeding techniques). Exploratory factor analysis (EFA) and confirmatory factor analysis (CFA) were performed to approve an acceptable fit of LATCH assessment with the five areas. Items with a high factor loading (i.e., λ value > 0.3) most accurately represented the proposed constructs. This step was a crucial procedure for achieving a global goodness-of-fit of the model before performing SEM on all the latent variables [44,45]. Secondly, the hypothesized SEM was tested to examine the relationships among the constructs. Thirdly, a multigroup analysis in the SEM was used to test the critical ratio (C.R.) for the differences between caesarean section and vaginal delivery groups estimates of breastfeeding techniques on exclusive breastfeeding initiation [44]. If the C.R. for differences between the two groups is between -1.96 to +1.96 using pairwise parameter comparison [44,46], we accept the null hypothesis (H_0_). If the C.R. is beyond -1.96 to +1.96, we reject the H_0_ [44,46].

Estimates of path coefficient represent the strength of the path between two variables, and were calculated using standardized regression coefficients (i.e., *β* value). The full information maximum likelihood (FIML) estimation method was used to estimate parameters most likely to represent population values [44]. To determine the suitability of model, several fit indices were used: the Goodness-of-fit Index (GFI), the Adjusted Goodness-of-fit Index (AGFI), the Incremental Fit Index (IFI), the Comparative Fit Index (CFI), and the Root Means Square Error of Approximation (RMSEA) [44,47]. The cutoff criteria for the fit indices were (1) GFI > 0.9, (2) AGFI > 0.9, (3) IFI > 0.9, (4) CFI > 0.9, and (5) RMSEA < 0.08 [48–50].

## Results

A total of 1,158 mother-infant dyads were invited from two postnatal wards, among which 952 women-infant dyads participated voluntarily in this study (response rate = 82.2%). The proportion of missing data was from 1.6 to 4.8% among the 10 items that were treated by mean substitution in our data analysis. The characteristics of the participants are summarized in Table 1. The ages of the women ranged from 17 to 46 years (mean = 30.81, SD = 4.66) and the ethnic composition of the women were Chinese = 34.4%, Malays = 23.3%, Indians = 23.3%, and others = 14.1%. The majority of the participants were multiparous mothers (60.1%) and had previously initiated exclusive breastfeeding (69.7%). The prevalence of cesarean section was 26.6% (N = 253) and the initiation rate of exclusive breastfeeding was significantly lower among the cesarean section group (58.9%) compared with vaginal delivery group (73.7%). Age, race, gestation, jaundice, comfort level (“C” subscale of LATCH assessment tool), degree of tears, and constipation were found to be significantly different between the cesarean section group and the vaginal delivery group (*P* < 0.05) using χ^2^ (Fisher exact test when expected frequency was less than 5) and independent *t* tests, respectively.

**Table 1:** Comparison of maternal characteristics, infant characteristics, breastfeeding technique, and breastfeeding initiation among cesarean section and vaginal delivery (N = 952).

	Entire sample	Cesarean section	Vaginal delivery	*P* value
	(N = 952)	(n = 253)	(n = 699)	
	n (%)	n (%)	n (%)	
Maternal characteristics				
Age [M (SD)]	30.81 (4.66)	32.17 (4.31)	30.32 (4.68)	<0.001^a^ ***
Race				
Chinese	371 (39.0)	87 (34.4)	284 (40.6)	<0.001^b^ ***
Malay	222 (23.3)	29 (11.5)	193 (27.6)	
Indian	222 (23.3)	94 (37.2)	128 (18.3)	
Others	137 (14.4)	43 (17.0)	94 (13.4)	
Parity				
Multiparous	494 (51.9)	131 (51.8)	363 (51.9)	0.967^a^
Primiparous	458 (48.1)	122 (48.2)	336 (48.1)	
Infant characteristics				
Gestation (d) [M (SD)]	272.41 (9.14)	270.57 (10.81)	273.08 (8.37)	0.001^a^ **
Birth weight (g)[M (SD)]	3114.59 (404.78)	3116.49 (442.14)	3113.90 (390.72)	0.953^a^
Jaundice				
Yes	271 (28.5)	88 (34.8)	183 (26.2)	0.009^b^ **
No	681 (71.5)	165 (65.2)	516 (73.8)	
Breastfeeding technique				
LATCH Assessment [M (SD)]				
L: Latch	1.86 (0.37)	1.87 (0.34)	1.86 (0.38)	0.504^a^
A: Audible swallowing	1.87 (0.38)	1.88 (0.39)	1.86 (0.37)	0.591^a^
T: Type of nipple	1.82 (0.42)	1.82 (0.42)	1.82 (0.42)	0.988^a^
C: Comfort	1.82 (0.42)	1.75 (0.50)	1.85 (0.39)	0.007**
H: Hold	1.69 (0.52)	1.67 (0.56)	1.69 (0.51)	0.525^a^
Total scores	9.06 (1.38)	8.99 (1.45)	9.08 (1.35)	0.390^a^
Exclusive breastfeeding initiation				
Yes	664 (69.7)	149 (58.9)	515 (73.7)	<0.001^b^ ***
No	288 (30.3)	104 (41.1)	184 (26.3)	
Postnatal complications				
Hypotonic uterine				
Yes	10 (1.1)	0 (0)	10 (1.4)	0.071^c^
No	942 (98.9)	253 (100)	689 (98.6)	
≥2^nd^ degree tears				
Yes	90 (9.5)	1 (0.4)	89 (12.7)	<0.001^c^ ***
No	862 (90.5)	252 (99.6)	610 (87.3)	
Bladder retention				
Yes	8 (0.8)	2 (0.8)	6 (0.9)	1.000^c^
No	944 (99.2)	251 (99.2)	693 (99.1)	
Constipation				
Yes	229 (24.1)	41 (16.2)	188 (26.9)	0.001^b^ **
No	723 (75.9)	212 (83.8)	511 (73.1)	
Hemorrhoids				
Yes	66 (6.9)	11 (4.3)	55 (7.9)	0.059^b^
No	886 (93.1)	242 (95.7)	644 (92.1)	

D = day; g = gram; M (SD) = Mean (standard deviation)

^a^ Independent t test

^b^ Chi-square test

^c^ Fisher’s Exact Test

** *P* < 0.01;

***P < 0.001

**Fig 2:**
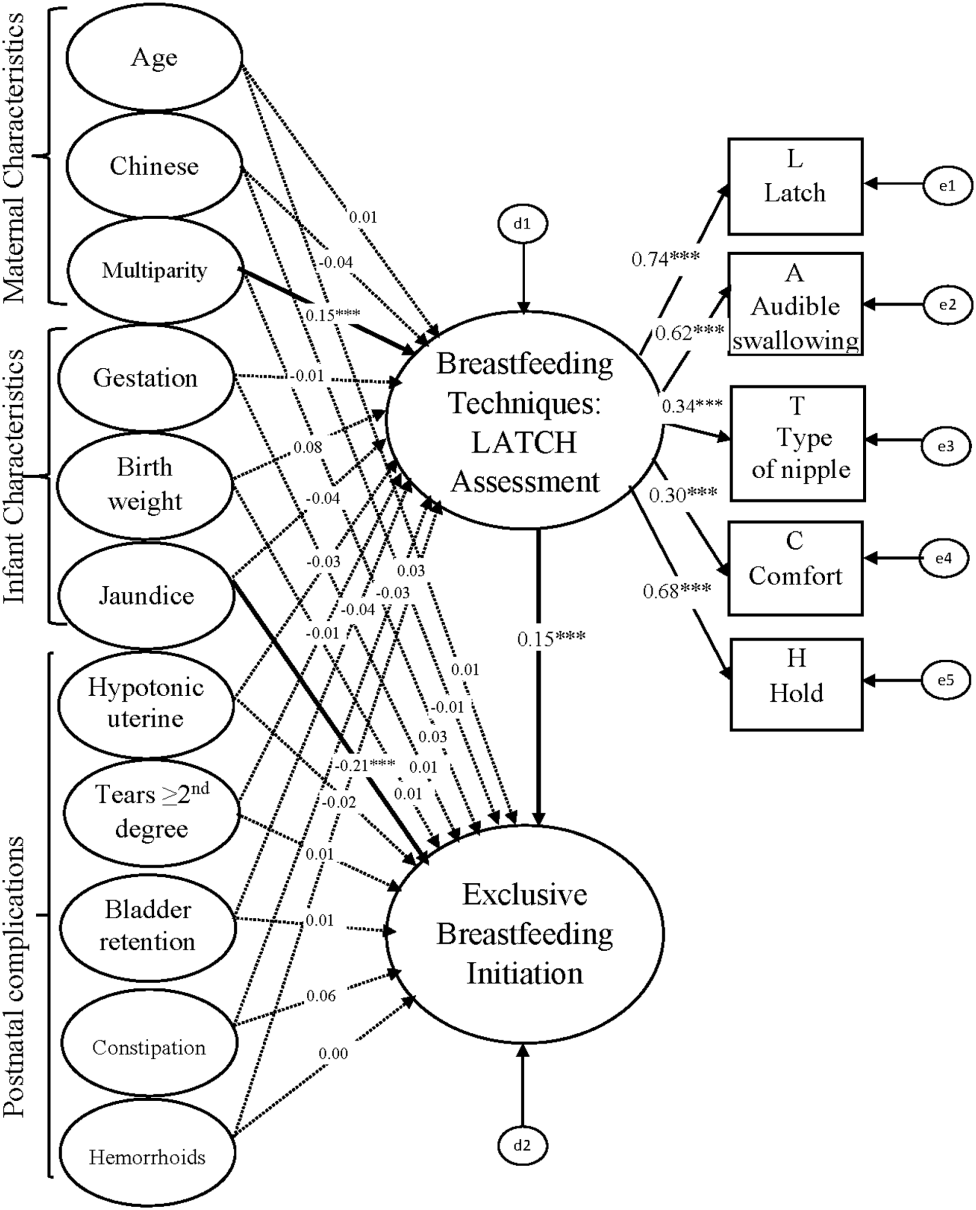
SEM model among entire population (n = 952).

In the first step, EFA and CFA were performed to confirm an acceptable fit of the LATCH assessment tool. The EFA revealed a one-factor structure for the LATCH assessment. The CFA model showed satisfactory fit indices (GFI = 0.989, AGFI = 0.966, IFI = 0.969, CFI = 0.969, and RMSEA = 0.069). In step two, the hypothesized SEM was tested to examine the relationships among the constructs. Fig 2 shows the evaluated full structural equation model with standardized coefficient and factor loadings. All the pathway estimates (*β* value) were reported in a standardized format. In step three, a multigroup analysis in the SEM was used to test the C.R. for differences between caesarean section and vaginal delivery groups estimates of breastfeeding techniques on exclusive breastfeeding initiation. The first structural equation model of the entire sample is reported (Fig 2). Model statistics indicated that the model fitted the data adequately (GFI = 0.987, AGFI = 0.962, IFI = 0.958, CFI = 0.955, and RMSEA = 0.034). Multiparity was significantly positively associated with breastfeeding technique (*β* = 0.15, *P* < 0.001) and the jaundice of an infant was significantly negatively associated with breastfeeding initiation (*β* = -0.21, *P* < 0.001). Breastfeeding techniques (*β* = 0.15, *P* < 0.001) were significantly positively associated with exclusive breastfeeding initiation in the entire sample. However, maternal age, maternal race, gestation, birth weight of infants, and postnatal complications had no significant impacts (*P* > 0.05) on breastfeeding techniques or exclusive breastfeeding initiation. All factor loadings (λ) for breastfeeding techniques ranged from 0.30 to 0.74 in the model.

**Fig 3:**
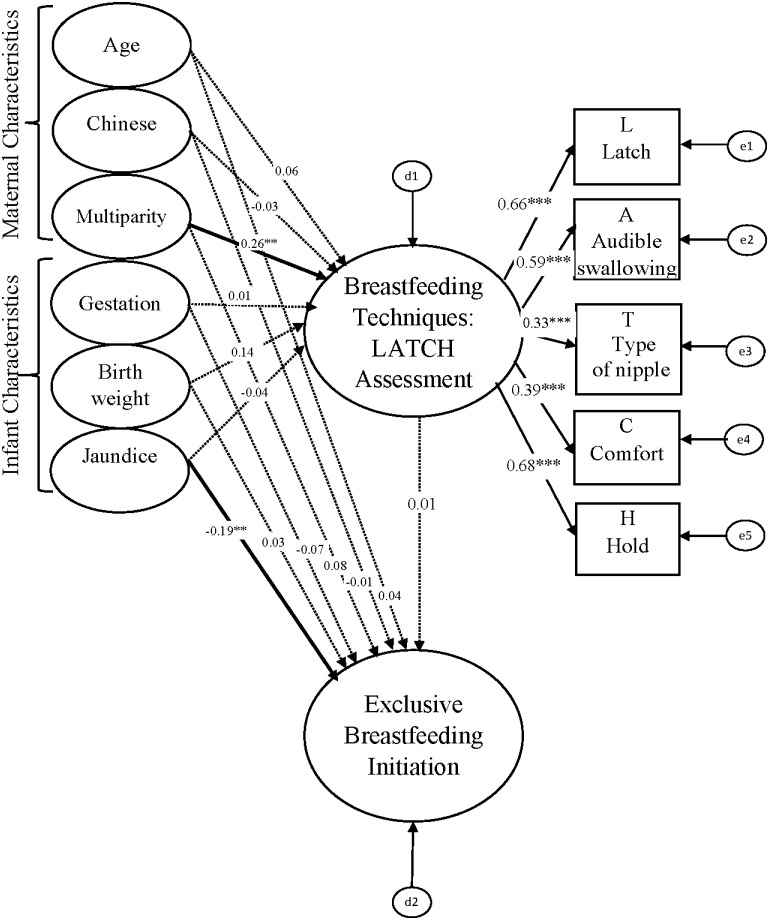
Multigroup analysis in the SEM among caesarean section group (n = 253).

**Fig 4:**
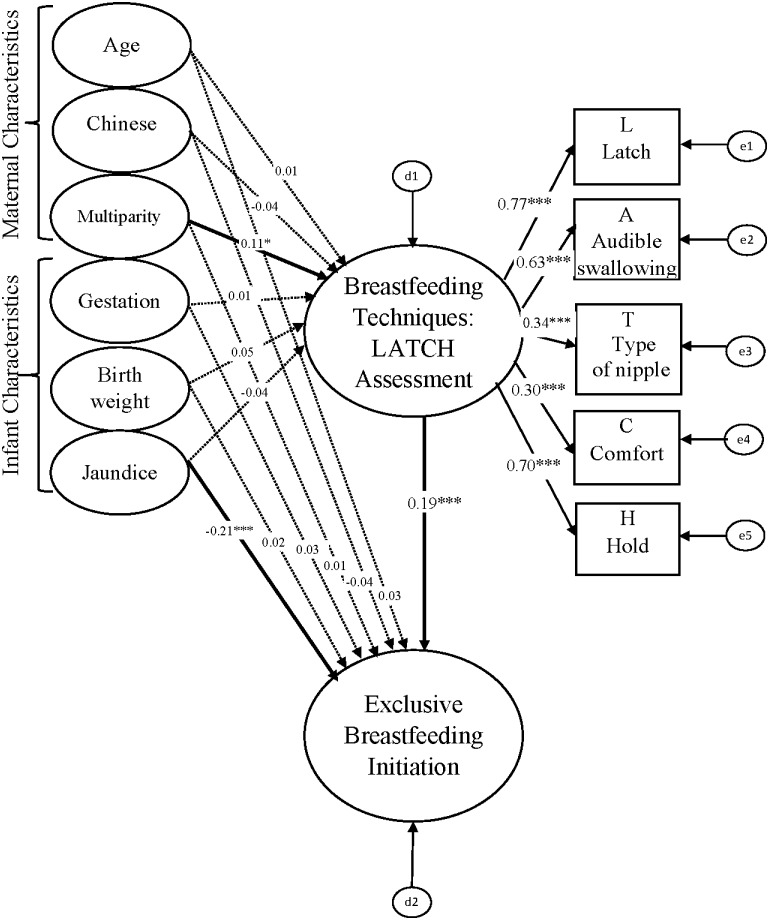
Multigroup analysis in the SEM among vaginal delivery group (n = 699).

The multigroup analysis tested critical ratio for differences between caesarean section and vaginal delivery groups estimates of breastfeeding techniques on exclusive breastfeeding initiation (Figs 3 and 4). Postnatal complications were not included in the multigroup analysis because sample size was not big enough for the SEM analysis in each separate group. The result of the C.R. for differences between caesarean section and vaginal delivery in this study was -1.429, so there was no difference between caesarean section and vaginal delivery groups estimates of breastfeeding techniques on exclusive breastfeeding initiation. Overall, the model fitted the data satisfactorily (GFI = 0.979, AGFI = 0.951, IFI = 0.962, CFI = 0.960, and RMSEA = 0.029). Multiparity was significantly positively associated with breastfeeding technique in the cesarean section group (*β* = 0.26, *P* < 0.01) and vaginal delivery group (*β* = 0.11, *P* < 0.05). Jaundice of an infant was significantly negatively associated with exclusive breastfeeding initiation in the cesarean section group (*β* = -0.19, *P* < 0.01) and vaginal delivery group (*β* = -0.21, *P* < 0.001). Breastfeeding technique was significantly positively associated with exclusive breastfeeding initiation in the vaginal delivery group (*β* = 0.19, *P* < 0.001) but not in the cesarean section group (*β* = 0.01, *P* > 0.05). All factor loadings (λ) for breastfeeding techniques ranged from 0.30 to 0.77 in these two SEMs. These results can be interpreted as a demonstration that the multiparity and jaundice of an infant are consistent factors for breastfeeding techniques and exclusive breastfeeding initiation respectively. The relationships between breastfeeding techniques and exclusive breastfeeding initiation are different between the cesarean section and vaginal delivery groups. However, maternal age, maternal race, gestation, and birth weight of infants had no significant impacts (*P* > 0.05) on breastfeeding techniques or exclusive breastfeeding initiation in the cesarean section and vaginal delivery groups.

## Discussion

To our knowledge, this is the first study to examine the relationships among maternal, infant characteristics, postnatal complications, breastfeeding techniques, and exclusive breastfeeding initiation using SEM approaches based on theoretical and empirical support. The prevalence of cesarean section was 26.6% in this study, which is similar to that of the region (i.e., Asia as a whole) [11] but is still higher than the global figure [9,10], far in excess of the optimal 15% recommended by WHO [8]. The relatively higher caesarean section rate in our finding highlighted the concern for appropriateness and necessity of performing cesarean section in Singapore. In conjunction with the evidence [7] that the exclusive breastfeeding initiation rate was significantly lower in the cesarean section group compared with the vaginal delivery group in this study, it is possible that the long duration of separation of the mother and infant due to the complications of cesarean surgery, such as pain, hemorrhage, and infections, has a negative impact on initiating breastfeeding exclusively [51]. Another possibility is that women may experience a long and difficult labor before cesarean section, all of which may delay the initiation of lactation [52]. In addition, women who choose to give birth by elective cesarean section may have lower inclination to breastfeed resulting from being less comfortable with the normal biological processes of child birth and breastfeeding [7].

This study has shown that breastfeeding techniques were related to exclusive breastfeeding initiation in the entire sample and in women who had vaginal deliveries, but not in those who had cesarean section. Our results partially support the hypothetical model that effective breastfeeding techniques led to exclusive breastfeeding initiation, which echoes the findings of previous studies. [31–33]. Effective breastfeeding techniques require good latch-on (baby grasps the breast well, mouth is wide open, tongue below the areola, lower lip upward, and suck with slow and deep movements) [32,33]. Audible swallowing appeared to be an important estimate of human milk intake in the early postpartum period because it is the gold standard for proof of milk transfer from the breast to the infant [31,33]. Correct positioning plays a crucial role in the establishment and maintenance of a good latch-on [31,33]. The findings support the recommendation that health-care professionals should be instructive to mothers’ breastfeeding techniques.

This study has shown that breastfeeding techniques were not related to exclusive breastfeeding in cesarean section group, the pattern of results appears to be consistent with previous studies [7,14,18]. Women who gave by caesarean session experienced a longer separation time between time and putting their baby to breast than women given birth vaginally [7], neuro-behavioral depression caused by labor analgesia may result in delay in exclusive breastfeeding initiation [14]. In addition, post-operative pain and limited mobility might interrupted breastfeeding initiation [18]. However, it is not clear why breastfeeding techniques did not influence exclusive breastfeeding initiation in caesarean section group; such issues require further studies to determine underlying causes.

When we compared maternal characteristics, infant characteristics, and breastfeeding techniques between the caesarean section and vaginal delivery groups, we found that older mothers, lower gestational age of infants, and lower comfort level of the LATCH assessment in the caesarean group compared to those in the vaginal delivery group. These evidence could alert health-care professionals to pay special attention to mothers with advanced age and infants with low gestational age during antenatal care who are in the high risk group for caesarean section. Moreover, it is necessary to implement active strategies, including gentle hand expression, warm compresses or shower prior to breastfeeding, frequently latching the baby, applying ice, using lanolin cream, and placing a silver spoon on the nipple between feeds [15,26] to improve the comfort level during breastfeeding in women who undergo cesarean section [53].

Consistent with a previous study [21], we found that mothers with more children were more likely to possess better breastfeeding techniques in the entire sample, and in both the cesarean section and vaginal delivery groups. Multiparous women were more likely to repeat their previous breastfeeding experiences and practices with their preceding children [54]. In contrast, first-time mothers who had little prior experience with infant feeding reported difficulties in handling their infants and in coordinating their movements during breastfeeding [15]. Furthermore, first-time mothers were less likely to be aware of WHO guidelines for breastfeeding [55] and were more likely to use pacifiers, which have been shown to be negatively associated with breastfeeding techniques [56]. Thus, supporting the first time mother should imply facilitation of the breastfeeding techniques during hospitalization.

Jaundice of an infant was less likely to initiate exclusive breastfeeding in the entire sample, in both the cesarean section and vaginal delivery groups, which consistent with a previous study [24]. A possible explanation might be that jaundiced infants may experience poor latch on and ineffective suckling [24]. One possible interpretation of this result is that the effects of phototherapy for infant jaundice that causes mother-infant separation and parental anxiety might interfere with exclusive breastfeeding initiation [57]. Another possibility is the maternal perception of insufficient milk production for their infants in first few days [58] because inadequate breastfeeding which leads to dehydration and / or starvation could increase jaundice [59]. Therefore, insecurity and guilty feeling of empty breasts left mothers with little confidence that they might be less likely to initiate exclusive breastfeeding [60]. Nevertheless, not-enough-breastfeeding jaundice and breastfeeding failure induced infant jaundice are largely preventable. Thus, a system-based approach [24,59] is needed to ensure that all infants receive skilled lactation support.

Maternal age, maternal race, gestation, and birth weight had no significant impacts on breastfeeding techniques or exclusive breastfeeding initiation in all the different modes of birth in our study. Since this is the first study designed to explore the relationship between maternal and infant characteristics, breastfeeding techniques, and exclusive breastfeeding initiation in the different modes of birth, it is impossible to do more than speculate as to why this may be the case at this stage. However, further investigation attempting to replicate these results with different populations is necessary before any firm conclusions can be drawn.

The LATCH assessment tool should be used in routine assessment and patient education. It emphasizes correct latching, watching for signs of swallowing, examining the types of nipple, improving comfort level, and positioning, i.e., the five important techniques for successful breastfeeding. Health-care professionals should also promote skin-to-skin contact between mothers and their infants shortly after cesarean section to improve breastfeeding initiation [61]. The first hours of skin-to-skin contact is critical for the development of the child’s thermoregulation and maternal sensitivity [62]. This is backed by previous research, which found that mothers who successfully initiate breastfeeding after cesarean section are as likely to practice exclusive breastfeeding at 6 months as mothers who give birth via vaginal delivery [7]. The findings of this study, taken together with those of previous studies, suggest the need for health facilities to implement protocols to increase the rate of exclusive breastfeeding initiation in early postpartum after cesarean section.

In addition, understanding population trends and determining the factors driving the rapid increase in cesarean section rates will provide valuable insights that will guide the development of programs [63] to reduce the numbers of non–medically indicated cesarean section. The difference in exclusive breastfeeding initiation among women who deliver via different modes highlights a need for hospitals to reorganize the way health-care professionals teach lactation, provide counseling, and support to women who breastfeed, and establish feeding and nutrition guidelines to support women who have undergone cesarean section. Increased monitoring of breastfed infants and mothers in the first 3 days of postpartum may allow early intervention to be initiated in order to facilitate successful initiation of lactation [64]. Nowadays, postnatal women are hospitalized for only 1 to 3 days compared to the past, where mothers remained in the hospital until breastfeeding had been successfully established. As such, health-care professionals have little opportunity to assess mothers while they are breastfeeding and to teach proper techniques, let alone being able to ascertain if a mother has successfully mastered the techniques of breastfeeding at the point of discharge from the hospital. Perhaps hospitals should implement or review existing post-discharge programs to support mothers in breastfeeding.

This study provides evidence that the link between maternal characteristics, infant characteristics, breastfeeding techniques, and exclusive breastfeeding initiation in the different modes of birth; however, the conclusions drawn should be considered in the context of its limitations. Firstly, the use of a cross-sectional design and convenient sampling method in a single setting may limit the generalizability of the results. Secondly, this research addressed only maternal characteristics, infant characteristics, and postnatal complications on breastfeeding techniques and initiation, but it did not address a number of other important factors such as maternal pre-pregnancy mass index, smoking status, previous breastfeeding experiences, belief, confidence, and social support. Thirdly, the incident neonatal jaundice was based on visual evaluation rather than serum bilirubin levels that can be the limiting accuracy of assessment. Fourthly, the components and scoring of the LATCH assessment tool was evaluated within 72 hours after delivery. Some may consider only the five parameters of breastfeeding techniques as too narrow and the time frame too short to be sufficiently sensitive for the tool to detect the differences between groups. Finally, exclusive breastfeeding initiation was measured as an outcome in this study which meant that we were unable to examine the breastfeeding duration over time, such issues require further studies.

## Conclusions

In sum, the SEM analyses conducted in this study have indicated adequate fit indices between the hypothetical model and the data. Clinically, the structural relationships would allow for effective assessment and management. The time to establish exclusive breastfeeding initiation in early postpartum is critical, as the mothers are in the midst of initiating breastfeeding. Hence, we need to make use of this chance to support the breastfeeding then the mother-infant dyads will have a lifetime beneficial effect. More research is needed to determine if maternal characteristics, infant characteristics, and postnatal complications other than the ones examined in this study affect breastfeeding techniques and initiation. Hopefully, these efforts will help more mothers breastfeed longer and more successfully.
